# Nano-substructured plasmonic pore arrays: a robust, low cost route to reproducible hierarchical structures extended across macroscopic dimensions†

**DOI:** 10.1039/d0na00527d

**Published:** 2020-08-11

**Authors:** Aurélien V. Gimenez, Kiang W. Kho, Tia E. Keyes

**Affiliations:** School of Chemical Sciences & National Centre for Sensor Research, Dublin City University Dublin 9 Ireland tia.keyes@dcu.ie

## Abstract

Plasmonic nanostructures are important across diverse applications from sensing to renewable energy. Periodic porous array structures are particularly attractive because such topography offers a means to encapsulate or capture solution phase species and combines both propagating and localised plasmonic modes offering versatile addressability. However, in analytical spectroscopic applications, periodic pore arrays have typically reported weaker plasmonic signal enhancement compared to particulate structures. This may be addressed by introducing additional nano-structuring into the array to promote plasmonic coupling that promotes electric field-enhancement, whilst retaining pore structure. Introducing nanoparticle structures into the pores is a useful means to promote such coupling. However, current approaches rely on either expensive top-down methods or on bottom-up methods that yield random particle placement and distribution. This report describes a low cost, top-down technique for preparation of nano-sub-structured plasmonic pore arrays in a highly reproducible manner that can be applied to build arrays extending over macroscopic areas of mm^2^ to cm^2^. The method exploits oxygen plasma etching, under controlled conditions, of the cavity encapsulated templating polystyrene (PS) spheres used to create the periodic array. Subsequent metal deposition leads to reproducible nano-structuring within the wells of the pore array, coined in-cavity nanoparticles (icNPs). This approach was demonstrated across periodic arrays with pore/sphere diameters ranging from 500 nm to 3 μm and reliably improved the plasmonic properties of the substrate across all array dimensions compared to analogous periodic arrays without the nano-structuring. The enhancement factors achieved for metal enhanced emission and surface enhanced Raman spectroscopy depended on the substrate dimensions, with the best performance achieved for nanostructured 2 μm diameter pore arrays, where a more than 10^4^ improvement over Surface Enhanced Raman Spectroscopy (SERS) and 200-fold improvement over Metal Enhanced Fluorescence (MEF) were observed for these substrates compared with analogous unmodified pore arrays. The experiments were supported by Finite-Difference Time-Domain (FDTD) calculations used to simulate the electric field distribution as a function of pore nano-structuring.

## Abstract 2

A simple approach to reproducibly nano-sub-structuring the cavity interiors in periodic pore arrays improves performance in surface enhanced Raman spectroscopy and metal enhanced fluorescence.

## Graphical abstract

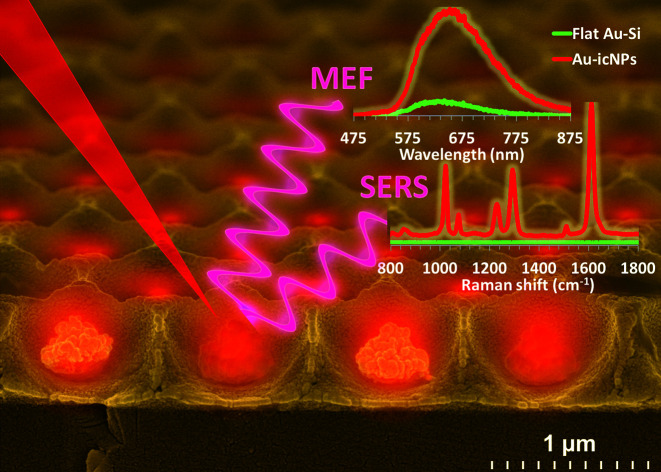

## 1 Introduction

Plasmonic materials are becoming widely important across diverse domains such as photochemistry,^1,2^ photocatalysis,^3,4^ optoelectronics^5^ and spectroscopy.^6–9^ And this diversity has been driven by advances both in nanochemistry/fabrication methods and in the theoretical understanding of plasmonic materials properties.^10,11^ Surface plasmons are quasi-particles arising from the collective oscillation of the free electron at a conductor–dielectric interface. They are most prevalent in coinage and noble metals due to their low losses at resonance frequencies (dissipation through scatter, inter-band transitions, *etc.*) and have been most widely explored in gold, silver and copper because their plasmon resonance is within the visible electromagnetic wavelength range. Localised surface plasmons that arise at nanostructured interfaces can be excited directly by incident light and, depending on the conductor material, the size and structure of the nanofeatures and the dielectric interface, intense local electric fields can be generated under plasmon excitation. Detection strategies that exploit spectroscopic signal enhancement at such plasmonic substrates are of growing importance in analytical and bioanalytical sciences.^12–14^ Such surface enhanced methods include, most notably, SERS and also surface-enhanced hyper-Raman scattering (SEHRS), MEF and surface enhanced infra-red absorption (SEIRA), and can offer dramatic signal enhancements yielding superior sensitivity to solution studies.^15^ Plasmonically enhanced spectroscopies require nano-structured substrates, particularly for vibrational spectroscopy, which can support intense localised plasmonic fields. The most dramatic SERS enhancements reported to date have been in aggregated nanoparticulate systems created using wet chemical methods, where single SERS molecule sensitivity has been achieved due to hotspots generated at nano-dimensional particle junctions.^16^ Correspondingly, the majority of substrates developed for SERS and related plasmonically enhanced spectroscopies rely on nanoparticulate structures, either free standing, including those with nanogaps contained within the structure, or immobilised 2-D arrays.^6,10,17–38^ The latter typically offer lower enhancements than aggregates, but significant advancements over recent years have led to routes with controlled, narrow interparticle separation leading to superior SERS enhancements and analytical performance in terms of reproducibility.^39^ However, the intensity of the electric fields at highly efficient hot spots can lead to incineration of the analyte, so a trade-off between the enhancement factor (EF) and substrate reproducibility is typically required in analytical applications.

While SERS substrates with the highest EF are particulate, for some applications, porous substrates are particularly advantageous and with careful engineering can offer high plasmonic fields.^40^ They offer (a) a volume into which the analyte solution can be entrapped; this is particularly useful in the context of fluorescence or in solution studies where the target can be isolated and concentrated at a surface on incubation from the analytical volume, *e.g.* with a capture surface,^41–44^ (b) a 3-dimensional volume that better matches the 3 dimensionality of the interrogation volume of most instrumental strategies, *e.g.* confocal microscopy volumes in Raman or fluorescence microscopy,^45^ and (c) continuous periodic array structures offering uniformity (low variance) in Raman and SERS intensity when spectra are collected from different sites across the periodic substrate. Ordered close packing facilitates a reliable understanding of the effects of incoming light and coupled propagating and localised cavity plasmonic modes and the associated tuning using excitation and detection angles and the height/depth of the voids can be deconvoluted from effects of disorder in the array structure which may affect relative contributions from the top surface and pore depending on structures under the laser focus in disordered structures.^46–51^ A number of approaches to prepare porous substrates have been reported^52–56^ including high cost electron beam and photolithographical methods. Alternatively, self-assembled plasmonic metallic nano-particles in various lattice formations can also be employed as a SERS active substrate.^57,58^ They can be fabricated with much lower cost, but often intrinsically less reproducible methods, including 2-dimensional sphere lithography.^59,60^ Low cost sphere array templating has been reported with vapour or electrodeposition of silver or most commonly gold, as described by Xu *et al.*^61^ or Bartlett *et al.*;^62^ the latter conducted an extensive study regarding the optical properties of such metallic nanocavity arrays.^59,63–67^

To augment the advantageous plasmonic fields of spherical void arrays, a number of approaches have been examined to improve both the electric field enhancement and the focusing of the field spatially within porous arrays. Zuo *et al.* demonstrated that creating inverted pyramidal rather than spherical shaped pore arrays improved SERS output in silver arrays and confined the electric field to mid-point in the volume.^68^

Bartlett and co-workers further showed that enhancement can be improved by placing nanoparticles (NPs) in the void which effectively roughens the surface. Theoretical calculations suggest that strong coupling arises between the NPs and the metallic void, the NPs further focusing the optical fields and concentrating light near the surface of the nano-voids.^69^ Experimental studies of Ag NPs and Ag NP aggregates in Au nanovoid arrays using colloidal solutions of metallic NPs show SERS and fluorescence signal enhancement compared to arrays without NPs.^70,71^ However, the authors report a large increase in the variability of the signal across the arrays due to the lack of control over the number of particles per void and/or their position at the surface between or inside cavities, which plays an important part in the magnitude of the enhancement. Without control of these elements, although the signal is increased, the analytical advantages of having a reproducible array are lost. While the fabrication of highly ordered Ag icNP structures was achieved by Xianglin Li *et al.*, the reported procedure was complicated by the need to skilfully transfer a polystyrene-sphere template between substrates.^72^ More recently, Malinovskis *et al.* reported an accessible alternative for the fabrication of plasmonic Au nanoparticle arrays deposited on nanoporous anodic alumina templates. However, the quality of the pore filling is limited by the size of the nanoparticles used, and the dimensions of the ordered region are limited by the anodization time during the fabrication process which is not extended above 10 h. The developed structures are potential substrates for SERS-based sensors but do not present a significant EF over nonporous anodic alumina templates without nanoparticles.^73^

To address these issues, as well as to improve fabrication throughput, we report here a simple and inexpensive approach to fabricate gold pore arrays with reliable and reproducible nano-sub-structuring confined to the bottom of the well. The approach is an add-on to conventional colloidal sphere templating that uses the template to generate the nanoparticulate structure. Localising the structure reproducibly within the well, in conjunction with a hexagonal packing configuration, can be used to modify the field intensity and SERS enhancement within the pore interior but in a controlled and reproducible manner. The strategy relies on previous observations that treatment of polymer with air or oxygen plasmas can lead to their nano-structuring.^74^ Here, we detail our fabrication method and the optimisation of the structure based on the SERS performance of the array. We demonstrate that our method can be applied reproducibly across different cavity/particle dimensions. Characterisation of the optimised substrates was performed by Field Emission Scanning Electron Microscopy (FESEM) and diffuse reflectance spectroscopy. And, along with FDTD simulations, the plasmonic properties of the nano-sub-structured arrays obtained by SERS and MEF measurements were compared to those of pore array substrates without encapsulated nanoparticles.

## 2 Results and discussion

**Scheme 1:**
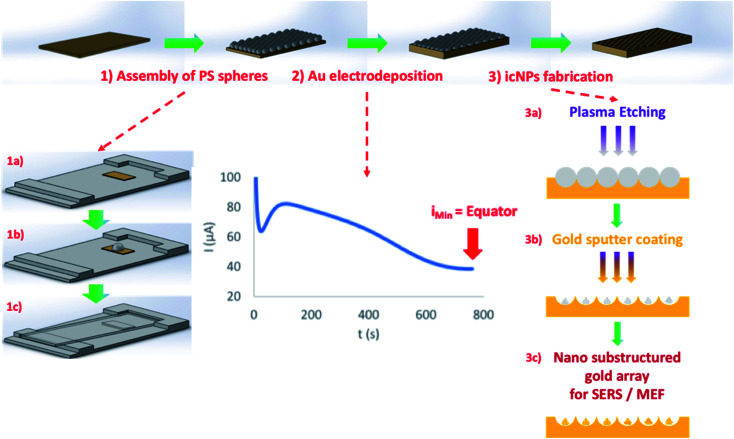
Schematic of the fabrication method of gold icNP arrays. (1) Assembly of a PS sphere monolayer in a hexagonally close packed arrangement on an Au–Si surface by gravity-assisted convective assembly (full details in the ESI†): the Au–Si substrate on an in-house deposition stage (1a), drop-casting of the PS sphere solution (1b), and convective drying of the solution using a glass slide (1c); (2) gold electrodeposition around spheres up to the equator; (3) icNP fabrication: plasma etching of trapped PS spheres (3a), followed by gold sputter coating (3b), leading to plasmonically active polymeric nano-substructures (3c).

The fabrication strategy leading to cavity-localised nanostructures is illustrated in Scheme 1.

Initial pore array substrate preparation was accomplished by iteratively optimizing a previously reported method for electrodeposition through a PS sphere template.^75^ The PS spheres were first assembled on a 100 nm thick gold layer deposited on an atomically flat silicon wafer to obtain a uniform, hexagonally close packed cm^2^ monolayer coverage of PS spheres on the Au–Si substrate. The full details for optimal close packed sphere assembly over cm^2^ areas are described in the ESI,† but in short, this is achieved by carefully drying the self-assembled spheres overnight at 4 °C on a slide with a tilted front angle of 2° and side angle of 1°. Au was then electrochemically deposited onto the underlying Au layer through the PS sphere template until a thickness equal to the radius of the spheres was reached. To ensure highly reproducible metal deposition, the amperometric *I*(*t*) curve for gold deposition on the PS sphere monolayer modified substrate was characterised by mapping the high-resolution Scanning Electron Microscopy (SEM) images to points along the *I*(*t*) curve to measure the gold deposition depth. Because of the reproducibility of the initial sphere assembly, the *I*(*t*) curve was also observed to be highly reproducible from substrate to substrate. From the *I*(*t*) curve a point was identified to reproducibly correspond to deposition to exactly 50% of the sphere height, *i.e.* gold deposition to the PS sphere equator. A representative *I*(*t*) curve, highlighting this 50% deposition point and the associated images of the cavity arrays are shown in the ESI.†

While the PS spheres remained in place in each well following electrodeposition, the polymer templates were then plasma etched using a polymer etching method adapted from the literature,^76,77^ leading to a reproducibly arranged nanoparticle in each pore. The etching conditions were investigated by iteration of experimental conditions and the optimised conditions were defined for each cavity/nanostructure size based on the best SERS enhancement efficiency and reproducibility of the final nanostructures as discussed *vide infra*. Following dry etching, substrates were sputter coated with gold to render the etched polymeric nano-substructures plasmonically active.

Preliminary investigations into the impact of radio frequency (RF) power, etching gas and flow ratio, and etching time on the morphology of 1 μm diameter PS spheres showed that RF power and etching time were the key parameters influencing the size and shape of the polymer nanostructure (see the ESI†).

Increasing the RF power from 100 W to 200 W resulted in more aggressive etching as larger sections of the polymer were removed from the initial PS spheres. Increased power might be used for faster etching particularly for larger spheres with more material to be etched. However, attempts to etch 3 μm diameter PS spheres at 200 W caused the outside of the sphere to melt smoothly making the harder core of the particle bloom rather like a flower of about 1.5 μm diameter on top of a water lily leaf measuring approximately 2.3 μm diameter (see the SEM image in the ESI†). These flattened structures were not particularly suited to SERS.

Indeed, at high RF power, the polymer is subjected to both chemical and physical etching. At low RF power, ions in the plasma have a low kinetic energy; therefore chemical etching is expected to dominate over physical etching. The reactive oxygen species, ions and radicals present within the plasma cause polymer strand cleavage leading to fragmentation of the PS into low-molecular-weight fragments.^78^ Maintaining a low RF power also prevents etching of the Au–Si substrate as the longitudinal etching rate remains low, confining etching principally to the polymer inside the cavity.

While a RF power of 100 W worked well for micrometre dimensioned spheres, it led to rapid etching of sub-micron spheres, with drastically diminished particle size from 510 nm to 160 nm within 6 minutes. For better control and more uniform etching, a gentler etching process was performed for 510 nm spheres where a RF power of 50 W was applied.

**Fig. 1:**
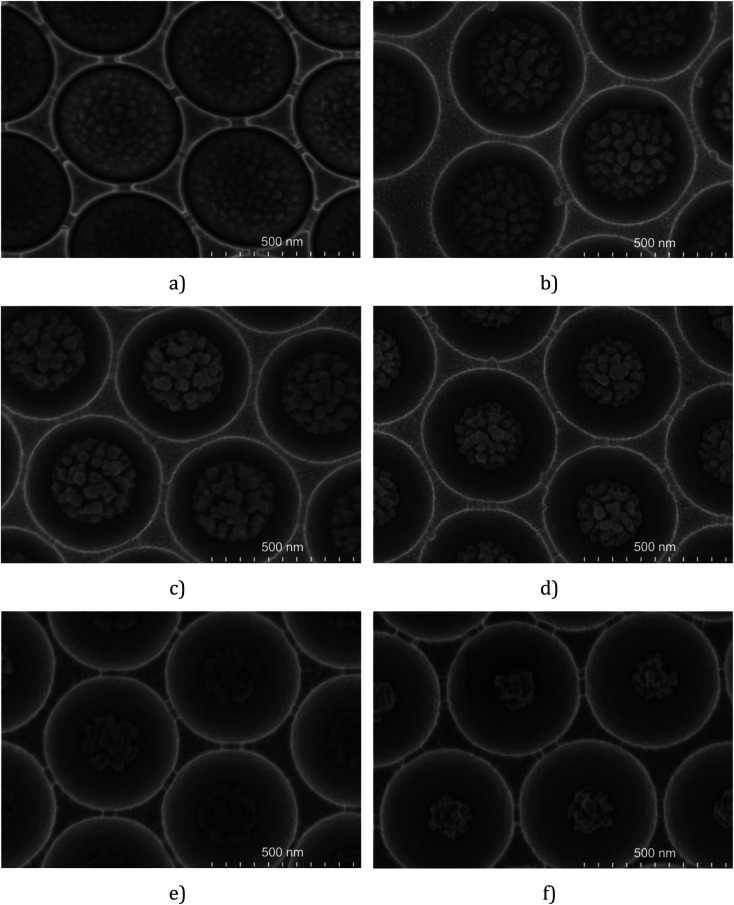
FESEM images of 510 nm diameter PS spheres in cavities etched for 4 min (a), 7 min (b), 8 min (c), 9 min (d), 11 min (e) and 14 min (f) (RF power = 50 W; *P* = 50 mTorr; O_2_ = 25 sccm).

Besides RF power, etching time also influences the size and shape of the final polymer particle. The results obtained for the etching of 510 nm PS spheres at 50 W are presented in Fig. 1 and show that the size of the PS decreases as the etching time increases (see the ESI†). The nanostructures etched onto the surface of the sphere remain at a constant size over the different etching times tested. But as time is extended the number of nanostructures decreases. And the size of in-cavity nanoparticles (icNPs) in each void is highly consistent across the substrate for a given etching time. Similar effects were reported by Yang *et al.*^77^ for 870 nm diameter PS spheres on planar surfaces.

For micrometre dimensioned spheres, we observed that extending the etching time increases the extent of roughening, leading to an agglomerate of PS strips. At short plasma exposure times, the small size of the etched polymer fragments allows for a uniformly roughened sphere. However, over extended etching times, the morphology of the PS spheres becomes more diverse, leading to reduced reproducibility from cavity to cavity.

To compromise between etching time and reproducibility of the morphology of the resulting structure, a RF power of 50 W was applied to etch 510 nm PS spheres and 100 W was applied for 1 μm, 2 μm and 3 μm ones. For each cavity size, the RF power, oxygen flow ratio and chamber pressure were fixed (values defined in the Experimental section) and the etching time was increased progressively. The SERS performance was then used to gauge the optimal etching conditions and is discussed in the section below.

To perform SERS each sample was coated with gold and functionalised overnight with a self-assembled monolayer (SAM) of 4,4′-bipyridine (4,4′-BPY) a SERS probe. Data were acquired at a minimum of 8 points across a single substrate and data from several substrates were included in the plot to reflect inter batch reproducibility and the continuity of the trends across multiple substrates prepared in the same manner.

The representative SERS spectra collected for 4,4′-BPY monolayers assembled across the optimised arrays are shown in Fig. 8. The spectra agree with those previously reported for 4,4′-BPY SERS, where the *ν*_8a_ mode at 1610 cm^−1^ attributed to ring C–C stretching (A_1_ ring mode) is the most enhanced feature.^79,80^ Here we used the *ν*_3_ inter-ring stretching (B_2_ ring) mode at 1293 cm^−1^ to estimate enhancement.^81–83^ This mode is the third most relatively enhanced feature of the 4,4′-BPY SERS spectrum compared to the Raman spectrum of solid 4,4′-BPY^80^ and was selected because it is well-isolated from any potential interference from the underlying PS.^84–86^

**Fig. 2:**
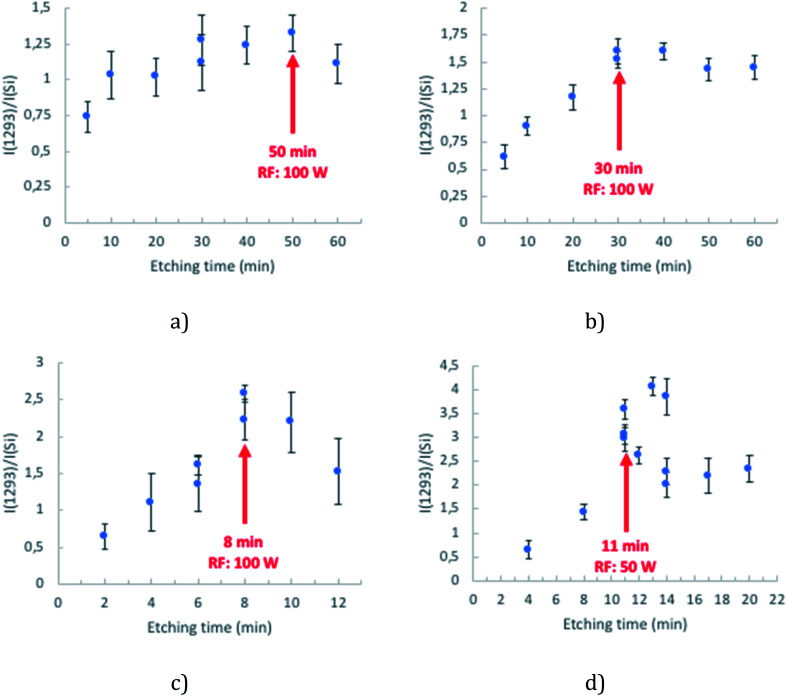
icNP development in 3 μm (a), 2 μm (b), 1 μm (c) and 510 nm (d) diameter cavities. The intensity reported corresponded to the B_2_ ring mode at 1293 cm^−1^ (*ν*_3_) and was normalised to the Raman signal of the Si standard measured on the day of analysis during the calibration of the equipment (*λ*_exc_ = 785 nm, 10 acc. 2 s, pin hole 500 μm, laser power at sample 429 μW, *n* = 8).

Fig. 2 presents the SERS spectra obtained across the different cavity sizes studied. Variability in SERS performance can originate from array preparation before the etching process. To demonstrate the remarkable uniformity in SERS performance across the array, data collected from completely separately prepared arrays are overlaid on a single plot.

The SERS enhancement for arrays of each given cavity diameter increases systematically with etching time until the SERS response plateaus or indeed, in the case of 1 μm spheres, decreases at longer plasma exposure times. A compromise between the maximum SERS intensity and minimum variability was thus identified and the optimal conditions of fabrication of the nano-substructured arrays are defined as 50 min, 30 min, 8 min and 11 min for 3 μm, 2 μm, 1 μm and 510 nm diameter cavity arrays.

**Fig. 3:**
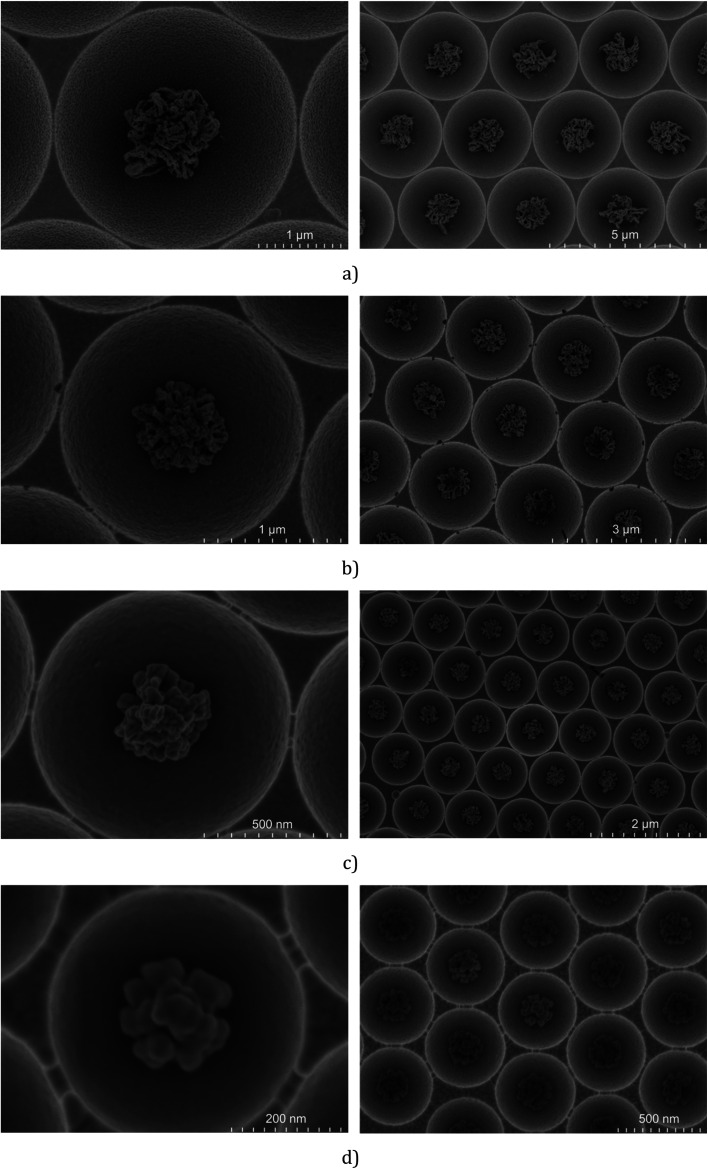
Representative FESEM images of optimised 3 μm (a), 2 μm (b), 1 μm (c) and 510 nm (d) diameter PS spheres in cavities etched, respectively, for 50 min, 30 min, and 8 min with a RF power of 100 W and 11 min with a RF power of 50 W.

The arrays prepared under the above defined conditions were characterised using FESEM. Fig. 3 shows representative FESEM images for the cavity encapsulated NPs in 3 μm, 2 μm, 1 μm and 510 nm diameter cavities.

The FESEM images show that the icNPs are morphologically and dimensionally very similar from cavity to cavity exhibiting long range homogeneity across the same substrate and also between substrates once preparation conditions are preserved. The dimensions of the icNPs vary only slightly from batch to batch with variation of only a few tens of nm.

It should be stressed that assembly of a highly uniform monolayer of PS and electrochemical deposition exactly to the hemisphere are essential pre-requisites to prevent discontinuity or multilayers in the spheres' arrangement and to obtain such a uniform distribution of icNPs. Indeed, since the fabrication method is based on a top-down approach, the absence of multi layers is crucial, as the upper-level layer of the polymer would essentially acts as a shield that impedes the etching rate of the subsequent layer underneath (see the ESI†).

**Fig. 4:**
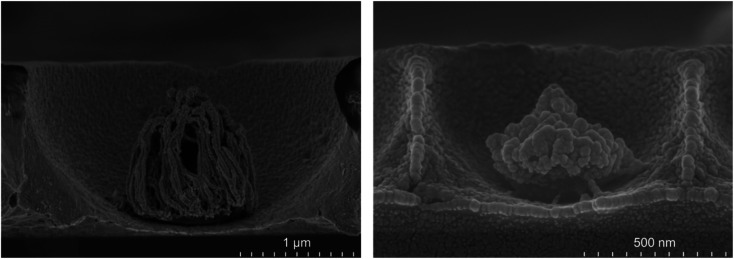
Side view FESEM images of a 3 μm diameter PS sphere in a cavity etched for 50 min with a RF power of 100 W (left) and a 1 μm diameter PS sphere in a cavity etched for 8 min with a RF power of 100 W.

Interestingly the morphology of the NPs varied reproducibly with the pre-etched dimensions of the templating spheres; larger PS spheres, those of 3 μm and 2 μm, form filament shapes under plasma etching, whereas for 1 μm and 510 nm diameter spheres the etching results in a cluster of spherical and pill shaped structures as illustrated in the FESEM side views presented in Fig. 4 (see the ESI†). Such PS sphere dependent topologies were reproducible over all replicates of each size of cavity arrays.

Correlating the morphology with SERS performance, we estimate that the best SERS enhancement is obtained when the ratio *η* (icNP diameter/cavity diameter) is approximately 0.4.

**Fig. 5:**
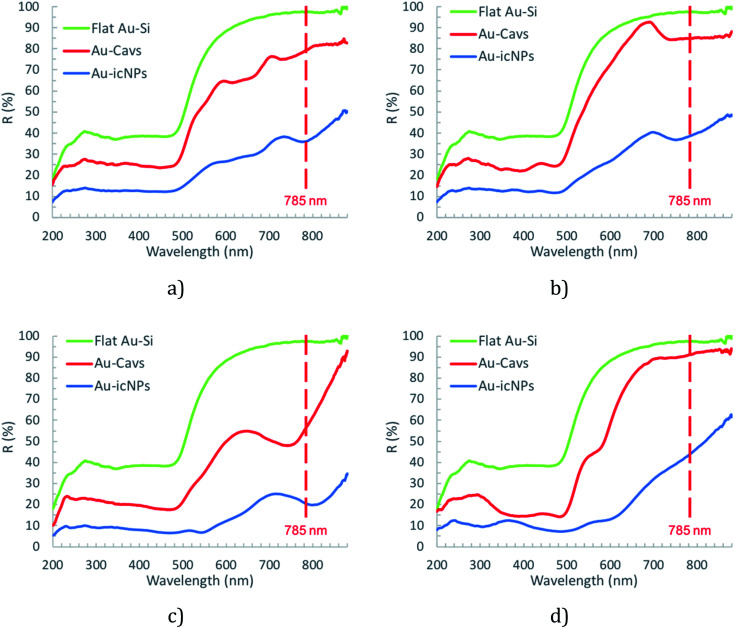
Diffuse reflectance spectra of dry flat gold (Au–Si) and cavity arrays without (Au-Cavs) and with icNPs (Au-icNPs) for different cavity diameters of (a) 3 μm, (b) 2 μm, (c) 1 μm, and (d) 510 nm.

To gain insight into their optical properties the optimised icNP modified arrays were characterised by diffuse reflectance spectroscopy. Cavity arrays without NPs (Au-Cavs) prepared under the same conditions of etching and sputtering were compared to icNP arrays (Au-icNPs) and the representative spectra are shown in Fig. 5.

The green line indicates the reference spectrum, which is the reflectance spectrum of gold electrodeposited onto a smooth Au–Si wafer. In all instances, reflectance decreases dramatically for pore arrays compared to planar gold and decreases significantly further, across the visible to near-infrared (NIR) optical window, for the nano-substructured substrates. This is expected as progressive nano-structuring is anticipated to increase absorbance due to localised surface plasmon modes.^87^ There is also a systematic shift to the red of the maximum absorbance for a given cavity size on introduction of nanostructuring but the change across the nanostructured arrays as a function of the underlying cavity size does not appear to be systematic, *i.e.* the Surface Plasmon Resonance (SPR) does not systematically shift to the red from large to small cavities with nanostructures. Specifically, for 510 nm diameter cavities, the lowest energy resonance shifts from 570 nm to 600 nm, for 1 μm diameter cavities it shifts from 745 nm to 800 nm, for 2 μm diameter cavities it shifts from 740 nm to 750 nm, and finally for 3 μm diameter cavities it shifts from 735 nm to 780 nm.

In addition, within the different sizes of nano-substructured substrates, one must consider the surface morphology of the icNPs, *i.e.* shape and roughness, which differs significantly for different particle sizes, as discussed above (*cf.*Fig. 3 and 4).

**Fig. 6:**
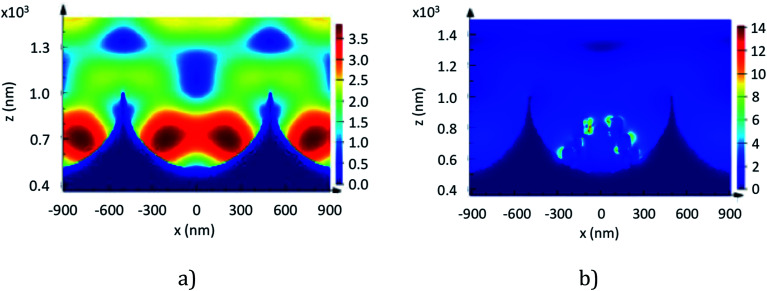
FDTD simulation of a 1 μm diameter cavity array without (a) and with (b) nanostructures under a 785 nm excitation laser at a normal incidence. Here, the field amplitude (right *y* axis) was not normalized and indicates the most intense value achieved in the nanostructure.

Remembering that the diffuse reflectance spectra contain contributions from both localised and propagating plasmons, the shift is influenced by three factors. The first, surface morphology, can be viewed as a series of inter-coupled plasmonic nanoparticles (see Fig. 6b).^88^ In general, this implies that the shift should increase with the increasing nano roughness. The second is the 3D geometry of the icNPs, *i.e.* the diameter and height of the structure influences the number of hot spots at the structure surface. Finally, the red shift can also be attributed to the electromagnetic coupling between the icNPs and the 2nd order surface propagating plasmon mode (^0^D in Cole *et al.*).^59,69^

FDTD simulation is a powerful tool to help understand the evolution and location of the electric field in the array. 1 μm diameter cavities were chosen for our numerical study as diffuse reflectance data presented previously show a plasmonic resonance peak for nano-substructured arrays close to 785 nm corresponding to the excitation line in our SERS experiment. To approximate the nanostructure, based on what we observe of its structure from FESEM characterisation, we simulate the structure as a superposition of elongated nano beads that form a pyramidal shape as a representation of the etched PS spheres (see Fig. 6b).

The simulated electromagnetic field structure within a 1 μm diameter cavity array with and without NPs, illuminated under normal incidence at 785 nm, is presented in Fig. 6. This figure allows comparison of the location and intensity of the electric field in the array. From the figure we observe that the field is predominantly located within the cavities whether NPs are present or not. For instance, for cavities without NPs, the two lobes near the bottom of the well exhibit an electric field amplitude that is about 50% higher than the field at the rim. Similarly, we can observe for cavities with icNPs that the hotspots are heterogeneously distributed and localised within the nanogaps at the icNP surface, and the amplitude of the electric field at the icNPs is about 700% higher than at the mouth of the cavity.

The electric field at the bottom of cavities with icNPs exhibits an approximately 4-fold increase in intensity, which corresponds well with the observed signal enhancement in our SERS and MEF experiments. By examining the simulated field structure, we could emphasise that aside from the amplitude of the electric field, other factors such as the size and number of hotspots also contribute to the apparent enhancement factor.

**Fig. 7:**
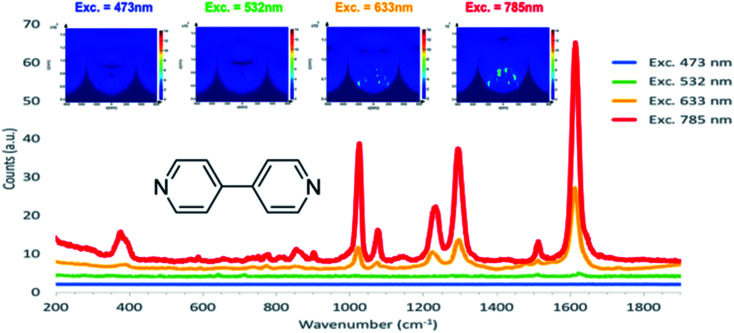
FDTD simulations of a 1 μm diameter cavity array with nanostructures under 473 nm, 532 nm, 633 nm and 785 nm excitation and the corresponding normalized SERS spectra of a 4,4′ BPY SAM functionalized at their surface.

Further FDTD simulations of 1 μm diameter nano-substructured arrays were carried out under three other excitation wavelengths (473 nm, 532 nm and 633 nm) and the corresponding SERS experimental data were acquired using 4,4′-BPY surface functionalised arrays. Fig. 7 shows simulations obtained for 473 nm, 532 nm, 633 nm and 785 nm excitation and the corresponding normalised SERS spectra. Simulations are displayed here with the same scale of electric field to enable interpretation of variation of intensities with excitation wavelength. As excitation shifts from blue to red, FDTD simulations show increasing field strength at the icNP's surface. Experimental data correlate with these predictions as the intensity of the SERS signal increases with red excitation, with the best SERS performance under a 785 nm excitation laser. This correlates well with the diffuse reflectance results shown in the previous section, where the plasmon absorbance maximum was found to be around 800 nm for such arrays.

Although FDTD simulations approximate the experimentally fabricated nano-substructured arrays, the presented simulations do match very well with the experimental data, suggesting that they reflect accurately the developed substrates.

The performance and signal reproducibility of arrays made through the optimised route were then evaluated for SERS performance whereby icNP arrays were compared to cavity arrays without NPs fabricated under identical experimental conditions.

This was achieved by preparing a cm^2^ array of the cavities of the desired diameter as described in the ESI.† Each sample was then split in half, with one half used as the cavity array without NPs once the PS spheres were washed out, and the second half was subjected to plasma etching of the spheres for icNP modification. To ensure as much analogy as possible between each array, each of the split arrays were etched and sputtered with gold at the same time, following the optimised parameters for the corresponding cavity size. Samples were then left to functionalise overnight to form a SAM of 4,4′-BPY for SERS characterisation.

**Fig. 8:**
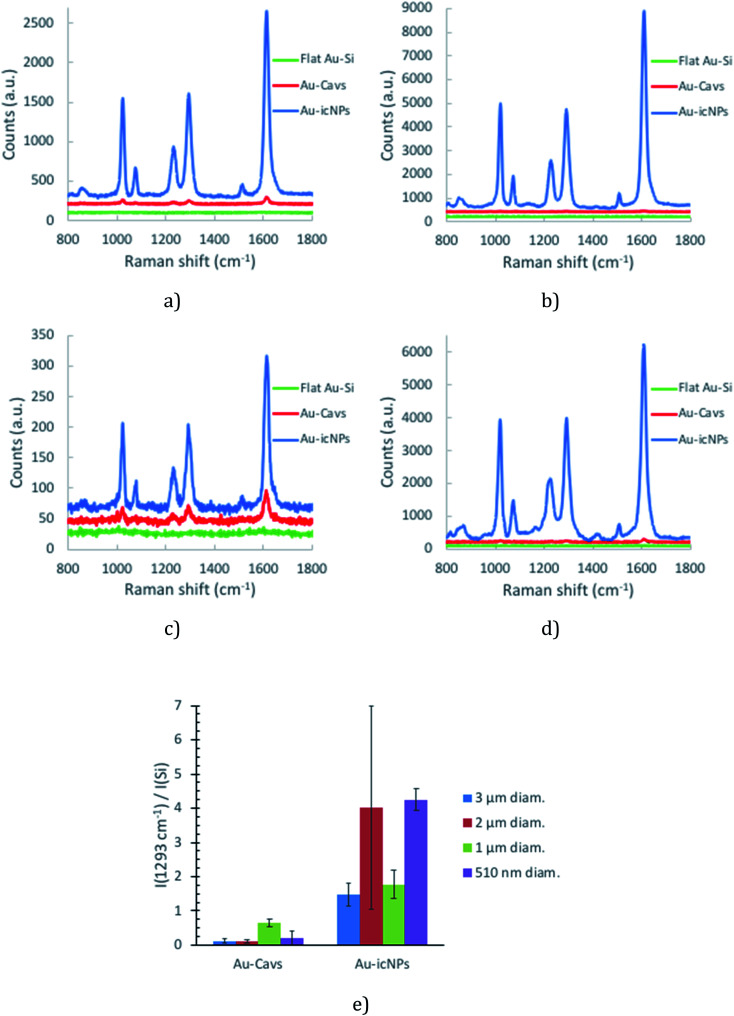
Representative Raman spectra for SERS measurements of a 4,4′-BPY SAM formed on 3 μm (a), 2 μm (b), 1 μm (c) and 510 nm (d) diameter cavity arrays with and without icNPs fabricated under analogous conditions. The data are as collected, background subtracted and unsmoothed (*λ*_exc_ = 785 nm, 10 acc. 2 s, pin hole 500 μm, laser power at sample 429 μW (a, b and d) and 94 μW (c)). A 10 mM ethanolic solution of 4,4′-BPY was used to functionalise the samples. (e) SERS intensity of the B_2_ ring mode at 1293 cm^−1^ (*ν*_3_) for the different samples tested.

This methodology was repeated for a minimum of three pairs of Au-Cavs/Au-icNP arrays obtained from completely independently fabricated batches. Each result presented below is an average of a minimum of eight SERS spectra acquired across the whole area of the tested array, to ensure the data is truly representative of the capability of each substrate. Fig. 8 gives the representative Raman spectra obtained for 3 μm (a), 2 μm (b), 1 μm (c) and 510 nm (d) diameter cavity arrays with and without icNPs, and Fig. 8e summarises the SERS performance and signal reproducibility across the four sizes of cavities studied, with and without icNPs.

Regardless of the cavity dimensions, the presence of NPs inside the wells consistently enhanced the SERS signal intensity compared to that of analogues without NPs. 3 μm diameter cavities with icNPs gave on average the lowest intensity of the SERS signal among the four types of nano-substructured arrays. 510 nm and 2 μm diameter cavities with icNPs gave on average the highest intensity of the SERS signal compared with their bare cavity array analogues. However, intra- and inter-sample variability differs with the dimensions of the substrates, reflected in the error bars shown.

Intra-sample measurements show a reasonable variability, which is acceptable considering the simple, low cost nature of the fabrication process. Variability across different sites of a single substrate ranges between 16% and 30% on average. Variability was the highest for 510 nm diameter cavities without NPs for which the intra-sample coefficient of variation (CV) was in some cases as high as 59%. The higher variability is likely due to the greater difficulty in controlling packing as uniform large array areas were more difficult to obtain with PS spheres below 1 μm diameter using the technique described in the ESI.† Furthermore, gold electrodeposition around the spheres is also likely to play a key role in reproducibility of the array. As the layer of gold is thinner for smaller diameter arrays, controlling the height of the cavities over the whole area of the substrate is more challenging.

Interestingly in comparison to the cavities without NPs, the presence of icNPs improves reproducibility of SERS performance for arrays of 3 μm and 510 nm diameter cavities as the inter-sample CV improves from 59% to 23% for 3 μm and from 111% to 8% for 510 nm diameter cavities. Such improvements illustrate the profound impact of the particles on the SERS intensity and confirm that the fabrication of the nanostructures within cavities is highly reproducible. The dramatic reduction in variability for the 3 μm and 510 nm diameter cavities strongly indicates that primary enhancement from the nano-substructured substrates arises from the particles in these cavity interiors. However, interestingly, the relatively low variability of 1 μm diameter cavity arrays without NPs of 18% is not improved by the presence of icNPs, which shows a comparable inter-sample CV of 23%. Finally, although the SERS signal is significantly enhanced for the icNP modified 2 μm diameter cavities, the bare and icNP substrates exhibited inter-sample CVs of 46% and 74%, respectively.

Based on these results, SERS enhancement factors (EF_SERS_) could be estimated for the different cavity dimensions with and without icNPs using eqn (1), where *I*_RS_ and *N*_RS_ are, respectively, the Raman signal intensity and the corresponding number of molecules for 4,4′-BPY in its solid form and *I*_SERS_ and *N*_SERS_ are the SERS signal intensity and the corresponding number of molecules on the SERS substrate's surface. The results are presented in Table 1. (The details of the EF_SERS_ calculation are given in the ESI.†)1
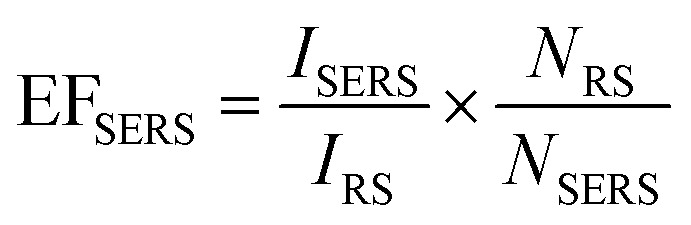


**Table 1:** EF_SERS_ obtained for different sizes of the Au cavity with and without icNPs using a 4,4′-BPY SAM as the SERS probe

Cavity diameter	EF_SERS_ Au-Cavs	EF_SERS_ Au-icNPs
3 μm	1.3 × 10^3^	1.6 × 10^4^
2 μm	1.2 × 10^3^	4.4 × 10^4^
1 μm	7.1 × 10^3^	1.9 × 10^4^
510 nm	2.2 × 10^3^	4.6 × 10^4^

Overall, the results presented highlight the advantages of incorporating nano-substructuring in the close packed cavity array in enhancing the SERS signal and in improving the reproducibility compared to cavities without NPs. The relative EF_SERS_ of Au-icNPs as compared to Au-Cavs ranges from ×3 (1 μm diameter cavities) to ×37 (2 μm diameter cavities). In addition, they ensure, unlike other approaches to nano-substructuring cavity arrays, that the particle and the hotspot are reliably positioned at the bottom of the well. Finally, the approach discussed can improve the variability of the SERS intensity measured from spot-to-spot and from substrate-to-substrate.

To assess the MEF properties of the arrays, we used the luminophore ruthenium-tris(2,2′-bipyridyl) dichloride ([Ru(bpy)_3_]Cl_2_). It has robust photochemical stability and relatively low quantum yield, which are required for a MEF probe. In the present context with emission around 630 nm, it corresponds reasonably well with the plasmon wavelength of the arrays and its large Stokes shift reduces the prospect of self-quenching. Furthermore, [Ru(bpy)_3_]Cl_2_ was used to probe cavity MEF in this manner in a previous report on related structures.^89^

500 nM [Ru(bpy)_3_]Cl_2_ in aqueous solution was used as a probe to assess the enhancement capabilities of the different sizes of cavity substrates with and without icNPs. The relative MEF enhancement factors (EF_MEF_) were compared between bulk solutions and across the different tested substrates, *i.e.* flat Au–Si and cavities with and without nanostructures, etched under similar conditions.

**Fig. 9:**
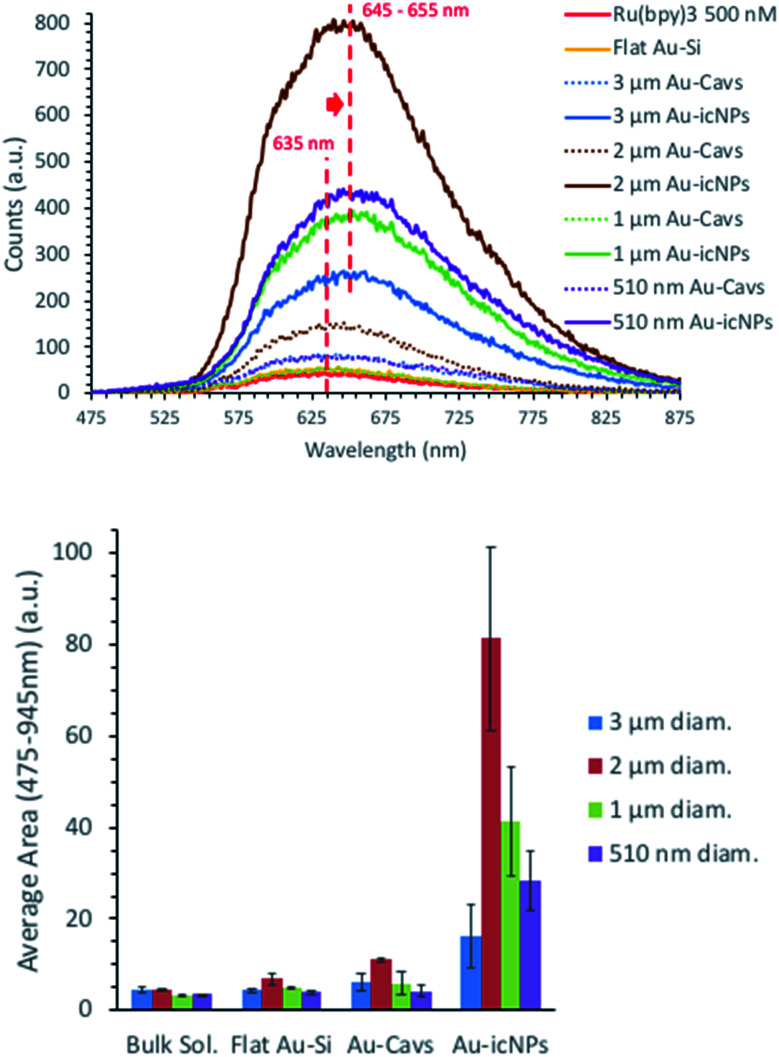
Emission measurement of 500 nM [Ru(bpy)_3_]Cl_2_ in bulk solution, over flat gold and within different sizes of cavities with and without icNPs fabricated under reproducibility conditions. (Top) Representative emission spectra; (bottom) area under the curve between 475 nm and 875 nm (*λ*_exc_ = 473 nm, 3 acc. 0.5 s, pin hole 500 μm, laser power at sample 57 μW).

Fig. 9 shows the emission spectra obtained for 500 nM [Ru(bpy)_3_]Cl_2_ filled 3 μm, 2 μm, 1 μm and 510 nm diameter cavity arrays with and without icNPs. As for the SERS data, each result presented below is an average of a minimum of eight emission spectra acquired across the whole substrate and of three samples fabricated in different etching batches. Also, all the results have been corrected for background in deionised water.

For each cavity dimension, a common trend can be described with the dye emission compared to the bulk solution increasing with the substrates' structural complexity, going from low enhancement with flat Au surfaces, progressively increasing with cavities without NPs and dramatically enhanced when icNPs are present.

The probe concentration is the same in all experiments and as the probe is not surface active, the MEF enhancement cannot be attributed to differences in the surface area between bare and particle containing cavities. Indeed, luminescence from molecules bonded or very close to the metal surface is expected to be quenched, and the number of molecules in the focal volume would actually be less for measurements on Au-icNP substrates *versus* measurement in bulk solution or Au-Cavs, and would be expected to be lower as a part of the substrate would physically occupy part of the confocal volume. Therefore, the luminescence enhancement can be confidently attributed to plasmonic enhancement hotspots at the micro/nanostructures.

The emission maximum of the luminescence spectra red shifts from 635 nm for the bulk solution to 645–655 nm for icNPs, further indicating emission intensity enhancement is MEF from NP hotspots.

EF_MEF_ at the different gold substrates studied was estimated by comparison to luminescence (*L*) obtained for the probe in bulk solution using eqn (2) and is given in Table 2. The EF_MEF_ presented here is expected to be slightly underestimated as described above because at the solid substrates we are not correcting for the space occupied by the substrate in the measured volume compared to the bulk solution.2
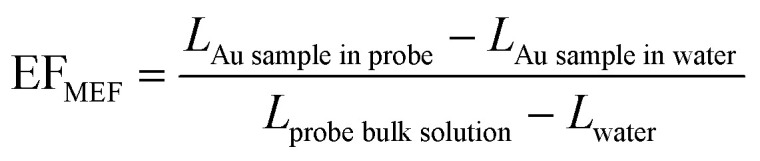


**Table 2:** EF_MEF_ obtained for flat gold and different sizes of Au cavities with and without icNPs using a 500 nM aqueous solution of [Ru(bpy)_3_]Cl_2_ as the luminescent probe

Cavity diameter	EF_MEF_ flat Au	EF_MEF_ Au-Cavs	EF_MEF_ Au-icNPs
3 μm	1.4	2.0	5.2
2 μm	1.5	2.4	17.9
1 μm	1.4	1.7	12.4
510 nm	1.1	1.2	8.1

The 2 μm diameter cavities gave best enhancement both without and with icNPs. Indeed, the emission signal corrected from the respective background was enhanced on average 2.4 and 17.9 times, respectively, for bare cavities and icNP substrates compared to bulk [Ru(bpy)_3_]Cl_2_, with enhancements up to a factor of 23 for the best performing icNP sample. Conversely, consistent with the Raman data, the 3 μm diameter cavities with icNPs were the least enhancing, with emission enhanced on average 5.2 times and the best performing sample giving up to a ×6.7 enhancement factor.

Although EF_MEF_ varies depending on the cavity/nano structure size, as mentioned, nano-substructuring the cavity array always leads to better enhancements, by up to more than one order of magnitude.

## 3 Conclusions

In conclusion, we have demonstrated a new and very simple low-cost and robust method to fabricate plasmonic nano-substructures within uniform gold cavity array substrates. Plasma etching of PS spheres within gold cavities proved to be a surprisingly reproducible method of nano modifying the arrays, producing pore arrays in which a highly structured nanoparticle is reliably localised at the bottom of the cavity. The method produces particles of very similar shape and size built both within a single substrate but also between batches of arrays. This technique has the advantage of being applicable over broad length scales from nano to micrometre sized spheres and leaves a nano-substructure at the bottom of every single cavity that presents multiple nano-defects with plasmonic activity once coated with gold. This has the effect of increasing and concurrently localising the intensity of the electric field to the nanoparticle at the bottom of the well, as shown by FDTD simulations. Using a 4,4′-BPY SAM and [Ru(bpy)_3_]Cl_2_, as SERS and MEF probes respectively, nano-substructured arrays were characterised and an increase in the signal by one to two orders of magnitude were observed for MEF and more than 4 orders of magnitude for SERS compared to their bare cavity equivalent, with nano structured 2 μm diameter cavity arrays showing the best overall enhancement. Overall, this low-tech, low cost approach to nanofabrication should be widely accessible and can provide tuneable plasmonic materials for a range of applications.

## 4 Methods

### 4.1 Fabrication of plasmonic nano substructures within gold cavity arrays

Gold micro and nanocavity icNP arrays were fabricated using sphere lithography followed by plasma etching as depicted in Scheme 1. Basically, PS spheres with diameters of 510 nm, 1 μm, 2 μm and 3 μm (Polysciences Inc.) were used to obtain different sizes of cavities/substructures. A large uniform assembly of a hexagonally close packed monolayer of polystyrene spheres was first formed on a gold-silicon wafer (AMS biotechnology, AU.1000.SL1) using a gravity-assisted convective assembly technique adapted from the literature (see the ESI†).^90,91^ Gold was then electrodeposited up to the equator of the spheres, ensuring the presence of a single particle centred in each well. The trapped PS spheres were physically modified by plasma etching using a RIE Plasmalab 80 Plus (Oxford Instruments). The optimal etching conditions based on the SERS signal for each cavity/nano structure size are presented in Table 3.

**Table 3:** Optimised etching parameters used for the different sizes of PS spheres during the fabrication of nanostructures in cavities

PS sphere size	RF power (W)	O_2_ flow ratio (sccm)	Chamber pressure (mTorr)	Etching time (min)
500 nm	50	25	50	11
1 μm	100			8
2 μm				30
3 μm				50

Following dry etching, the substrates were sputter coated with gold for 30 s using a current of 30 mA (Sputter coater Model 108, Cressington), leading to an averaged film thickness of 37 nm rendering the etched polymeric nano-substructures plasmonically active.

### 4.2 Characterisation of the plasmonic nano-substructured gold substrates

#### 4.2.1 SEM and FESEM characterisation

SEM images were collected using a Hitachi S3400n SEM tungsten system instrument. FESEM images were obtained using a Hitachi S5500 cold field emission in lens Scanning Electron Microscope ensuring high resolution images of the nano-substructures. All images were acquired using the secondary electron mode.

#### 4.2.2 Diffuse reflectance

Diffuse reflectance measurements were carried out in air using a Jasco V-670 Spectrophotometer with a 60 mm diameter integrating sphere Jasco ISN-723. Spectra were acquired between 200 and 880 nm using Spectra Manager software. Aiming at focusing the light at the close packed cavity array and limiting exposure of the surrounding flat gold areas, a black mask (black paper) with a 4 mm diameter window was applied on the measured samples. Background spectra were collected using Spectralon® as a standard.

### 4.3 FDTD simulations

FDTD simulations were performed for 1 μm diameter bare and nano-substructured cavity arrays using Lumerical software. A macro was written to generate an assembly of pill shape structures, reducing their number for each successive layer until reaching the desired height of the nanostructure. To emulate as closely as possible the structures from FESEM characterisation, randomness in the aggregation was introduced in the simulation code to render each NP unique. Only the shell of the nano pills was simulated as gold, with a varying thickness of the film up to 36 nm. For this study, only arrays with 1 μm diameter cavities were simulated. The optical constant for Au was obtained from Johnson & Christy.^92^ As the array contains a large contrast between feature sizes (*e.g.* the inter-cavity gap-size is more than 30 times smaller than the cavity diameter), conformal variant 2 was used as the mesh refinement method to ensure simulation accuracy. Simulations were performed with a resolution of 8 nm. For field calculations, the illumination laser bandwidth was set at 0.02 nm. The central wavelengths used in the simulations are 473 nm, 532 nm, 633 nm and 785 nm. All simulations were terminated at an auto shutoff threshold of 10^−5^. To simulate an infinitely large periodic structure, Bloch boundary conditions were used.

### 4.4 SERS/MEF

#### 4.4.1 Preparation of substrates for SERS characterisation

To evaluate the SERS properties of the fabricated arrays, samples were dipped overnight in a 10 mM ethanolic solution of 4,4′-BPY. To ensure that physisorbed molecules do not contribute to the SERS signal, samples were rinsed with a large volume of ethanol and, without letting the sample dry, successively rinsed with a copious amount of deionised water to make sure no ethanol remained in the cavities. The water-filled cavity arrays were then placed in a reusable microfluidic device as illustrated in the ESI† and SERS spectra were acquired in water.

#### 4.4.2 Preparation of substrates for MEF characterisation

Dry substrates were first dipped in ethanol to allow pre-wetting and subsequently rinsed with a copious amount of deionised water to make sure no ethanol remained in the cavities. Wet substrates were then rinsed with a 500 nM [Ru(bpy)_3_]Cl_2_ aqueous solution and allowed to sit in it before being placed in a reusable microfluidic device as illustrated in the ESI,† which was filled with the probe solution for emission spectrum acquisition.

#### 4.4.3 SERS and MEF measurements

SERS and fluorescence spectroscopy measurements were carried out on a Labram HR instrument (Horiba) with a 50× (0.55 N.A.) long distance magnification objective (Leica). The wavelengths of different laser sources used for excitation were 473 nm, 532 nm, 633 nm and 785 nm presenting, respectively, a full power at the sample of 5.03 mW, 1.02 mW, 6.00 mW and 60.2 mW. Neutral density filters were applied to reduce the intensity of the laser and the corresponding power at the sample is given with the acquisition conditions in the presented figures. SERS and fluorescence signals recorded were normalised to the Raman signal measured for the Si peak on the day of analysis during the calibration of the equipment.

Fluorescence measurements were carried out using a 473 nm excitation wavelength to excite the luminophore [Ru(bpy)_3_]Cl_2_ chosen as a probe to assess the MEF properties of the arrays.

## Conflicts of interest

There are no conflicts to declare.

## Supplementary Material

NA-002-D0NA00527D-s001
