# Supplementary material for: Nano-substructured plasmonic pore arrays: a robust, low cost route to reproducible hierarchical structures extended across macroscopic dimensions
Source: Nanoscale Adv. 2020 Aug 11;2(10):4740–56. doi: 10.1039/d0na00527d (PMC9417107; doi:10.1039/d0na00527d)
Supplement: NA-002-D0NA00527D-s001 [file NA-002-D0NA00527D-s001.pdf]

Electronic Supplementary Material (ESI) for Nanoscale Advances.
This journal is © The Royal Society of Chemistry 2020

Electronic Supplementary Information (ESI)

Nano-substructured Plasmonic Pore Arrays: A robust, low cost route to reproducible hierarchal structures extended across macroscopic dimensions.

Aurélien V. Gimenez, Kiang W. Kho, Tia E. Keyes
School of Chemical Sciences & National Centre for Sensor Research, Dublin City University, Dublin 9, Ireland.

Contents

1 Easy and robust fabrication of large uniform well-ordered gold nano and micro cavity arrays for SERS applications. 2
1.1 Large uniform assembly of a hexagonally close packed monolayer of polystyrene spheres onto a gold-silicon wafer. 2
1.2 Fabrication of hemisphere gold cavities by controlled electrodeposition up to the equator. 3
2 Influence of etching process parameters on resulting NP morphology 6
2.1 Etching 1 µm diameter PS sphere varying etching parameters 6
2.2 Etching 3 µm diameter PS sphere with RF Power of 200 W 7
2.3 icNP diameter vs. etching time during etching of 510 nm diameter PS sphere with RF Power of 50 W 7
3 Importance of a highly uniform monolayer PS sphere for a uniform distribution of icNPs 8
3.1 Defects created when etching multilayer of polymer 8
3.2 Tilted view of icNPs arrays after etching a highly uniform monolayer of PS spheres 8
4 SERS enhancement factor detailed calculation 8
5 Reusable microfluidic device for SERS and MEF application 10
6 References 11

1 Easy and robust fabrication of large uniform well-ordered gold nano and micro cavity arrays for SERS applications.

1.1 Large uniform assembly of a hexagonally close packed monolayer of polystyrene spheres onto a gold-silicon wafer.

A 1.5 cm by 0.8 cm Au-Si sample was cut, cleaned with acetone, water and ethanol, dried, then air plasma treated for 5 minutes before being placed on a deposition stage fabricated for this purpose. 20 µL of a 1 % (solid wt. in solution) PS spheres solution was drop-cast on the Au-Si substrate. A cleaned glass slide was placed over the sample forming a 2 ° angle with the substrate base. The deposition stage was left to dry overnight in the presence of silica-gel at a temperature of 4 °C, with a tilted front angle of 2 ° and side angle of 1 °. Once the water had fully evaporated, the Au-Si substrate shows an iridescent deposit indicative of a close packed monolayer of dry PS spheres (see Figure S 2). Figure S 1 illustrates the different steps leading to the hexagonal close packed monolayer assembly onto the Au-Si substrate.

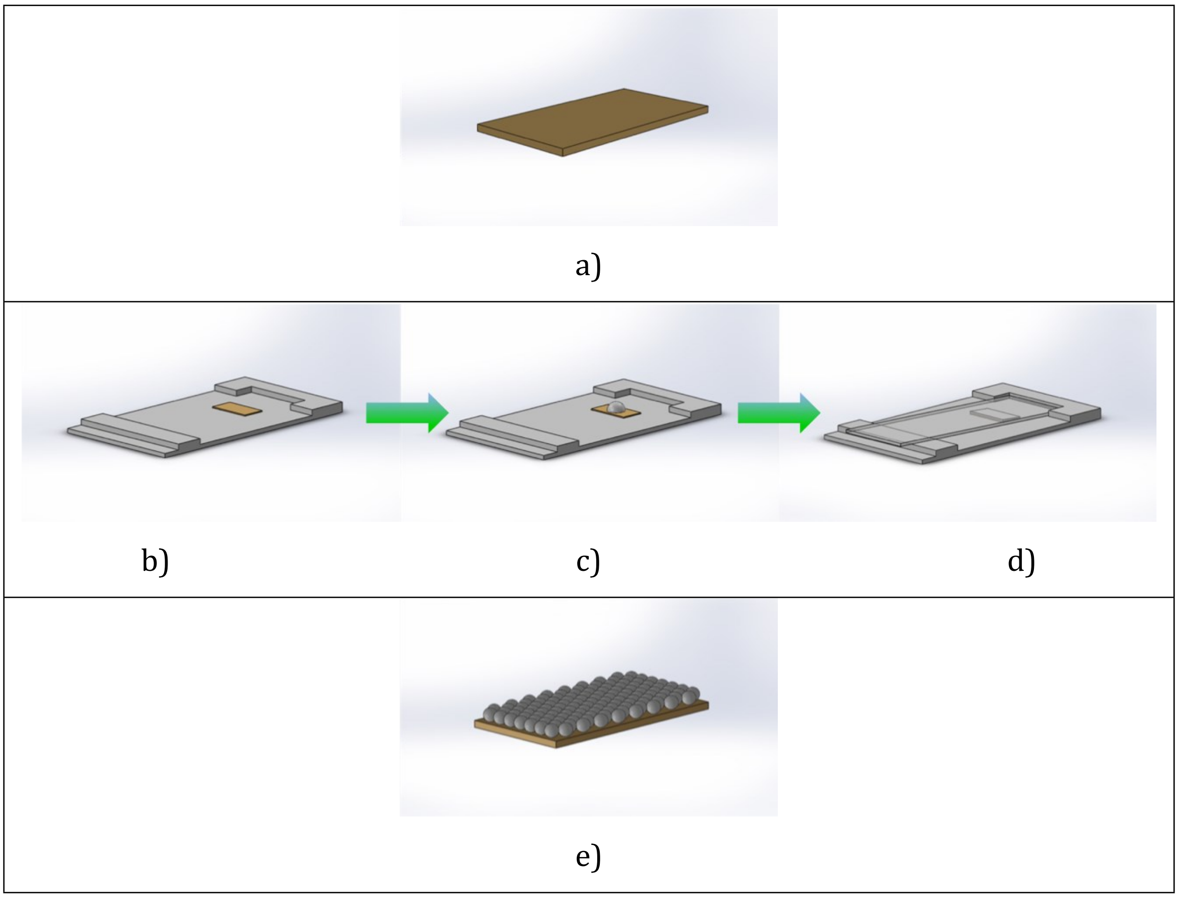

Figure S 1: Schematic of the fabrication method of a PS spheres hexagonally close packed monolayer onto an Au-Si wafer by convective assembly a) Clean 0.8 x 1.5 cm Au-Si substrate, b) Substrate stuck onto deposition stage, c) 20 µL of a 1 % (solid wt. in solution) PS spheres drop cast onto Au-Si substrate, d) Glass slide placed over sample forming a 2 ° angle with the substrate, e) Monolayer assembled at the Au-Si surface in a hexagonally close packed arrangement

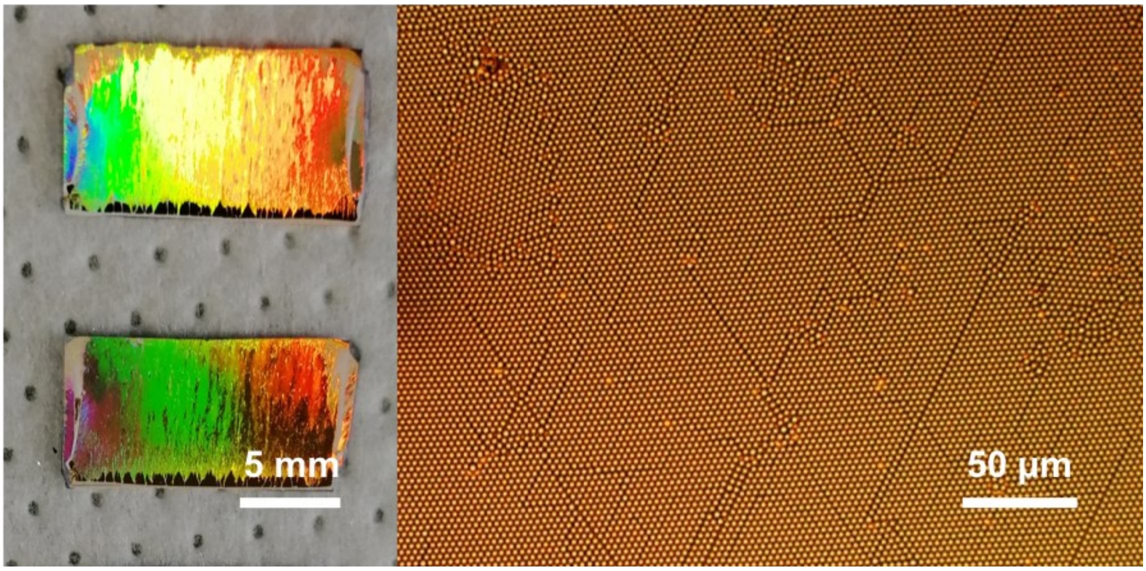

Figure S 2 : (Left) Picture of two 1.2 cm² gold-silicon samples on which was assembled a 3 µm diameter PS spheres monolayer following the most optimized version of the method described previously. Samples are presenting an iridescent surface characteristic of well-organized hexagonally packed assembly of the spheres. (Right) White-light microscope image of the same sample captured with a 20x objective.

1.2 Fabrication of hemisphere gold cavities by controlled electrodeposition up to the equator.

Once the PS spheres monolayer has been assembled at the Au-Si surface, gold electrodeposition through the spheres template was performed. The electrochemical cell consists in the Au-Si wafer being the working electrode, a Ag/AgCl reference electrode and a platinum wire as counter electrode, linked to a potentiostat CH Instrument model 900 electrochemical station.

Electroplating gold solution Elevate gold 7990 RTU (8.2 g/L) was purchased from Technic Inc. USA. As slight variations might occur from batch to batch, best electrodeposition potential needs to be defined and was found here to be -600 mV. The solution degassed by purging for 30 minutes with nitrogen. The I(t) curve was monitored, and gold electroplating was stopped when current reached the minimum corresponding to the equator level of the spheres monolayer as illustrated in Figure S 3 and Figure S 4. Sample were finally rinsed with deionised water avoiding salt to crystallise at the gold surface and were dried under nitrogen stream.

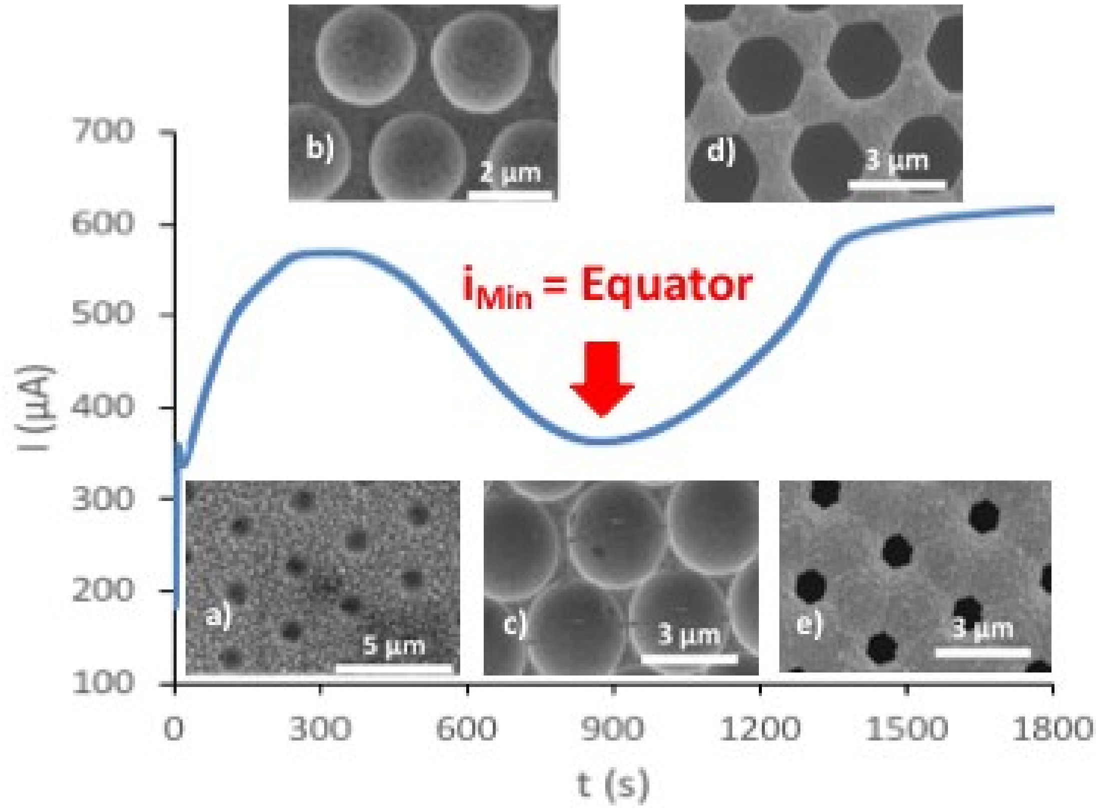

Figure S 3 : Representative I(t) curve obtained during the electrodeposition of gold around 3 µm diameter hexagonal close packed PS spheres monolayer assembled onto a Au/Si substrate. (E = -0.6 V vs. Ag/AgCl). Representative SEM images of resulting gold cavity arrays when stopping the electrodeposition before reaching the equator (a) and b)), at the equator (c) and passed the equator (d) and e)).

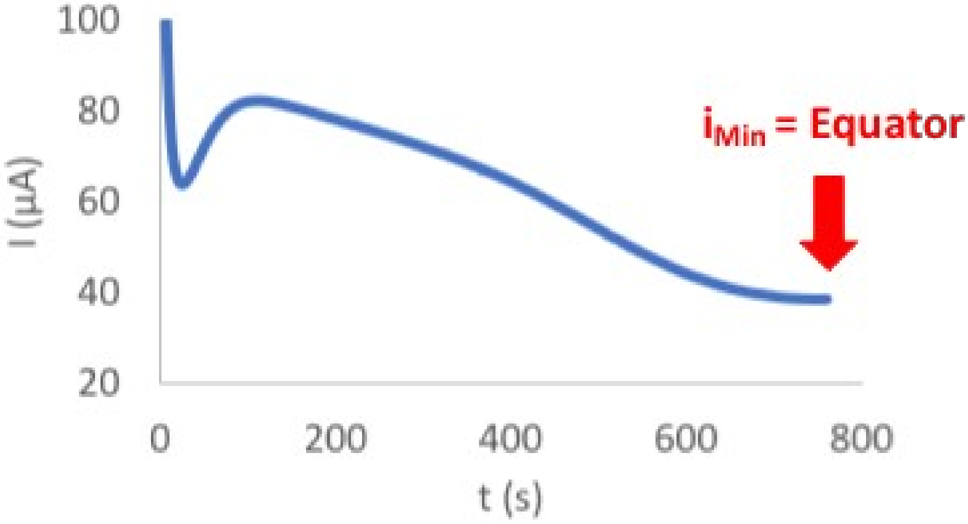

Figure S 4 : Representative I(t) curve obtained during the electrodeposition of gold around 1 µm diameter hexagonal close packed PS spheres monolayer assembled onto a Au/Si substrate, up to a depth of the equator of the spheres. (E = -0.6 V vs. Ag/AgCl).

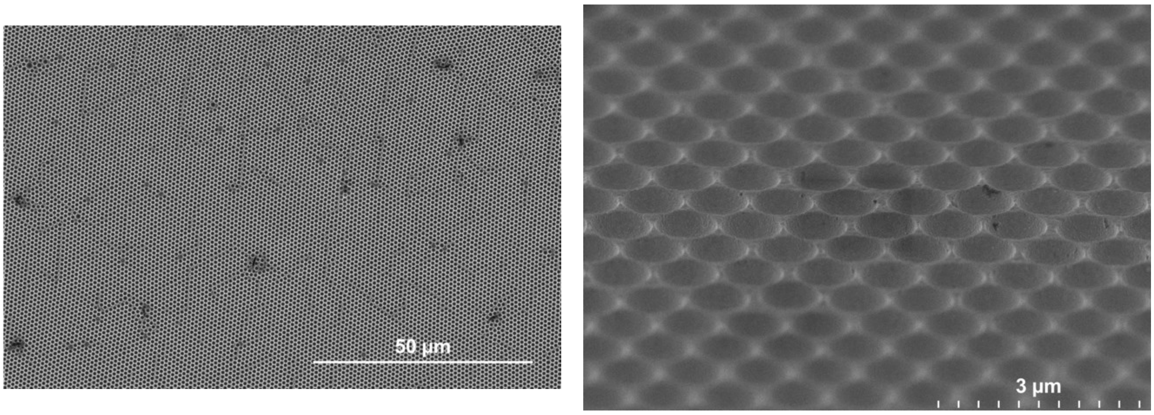

Figure S 5 : Representative FESEM images of a large uniform hexagonally close packed 1 µm diameter gold cavity arrays grown until the equator of the spheres' monolayer and after spheres removal by dissolution of the polymer in THF. (Left) Top view; (Right) Tilted view.

2 Influence of etching process parameters on resulting NP morphology

2.1 Etching 1 µm diameter PS sphere varying etching parameters

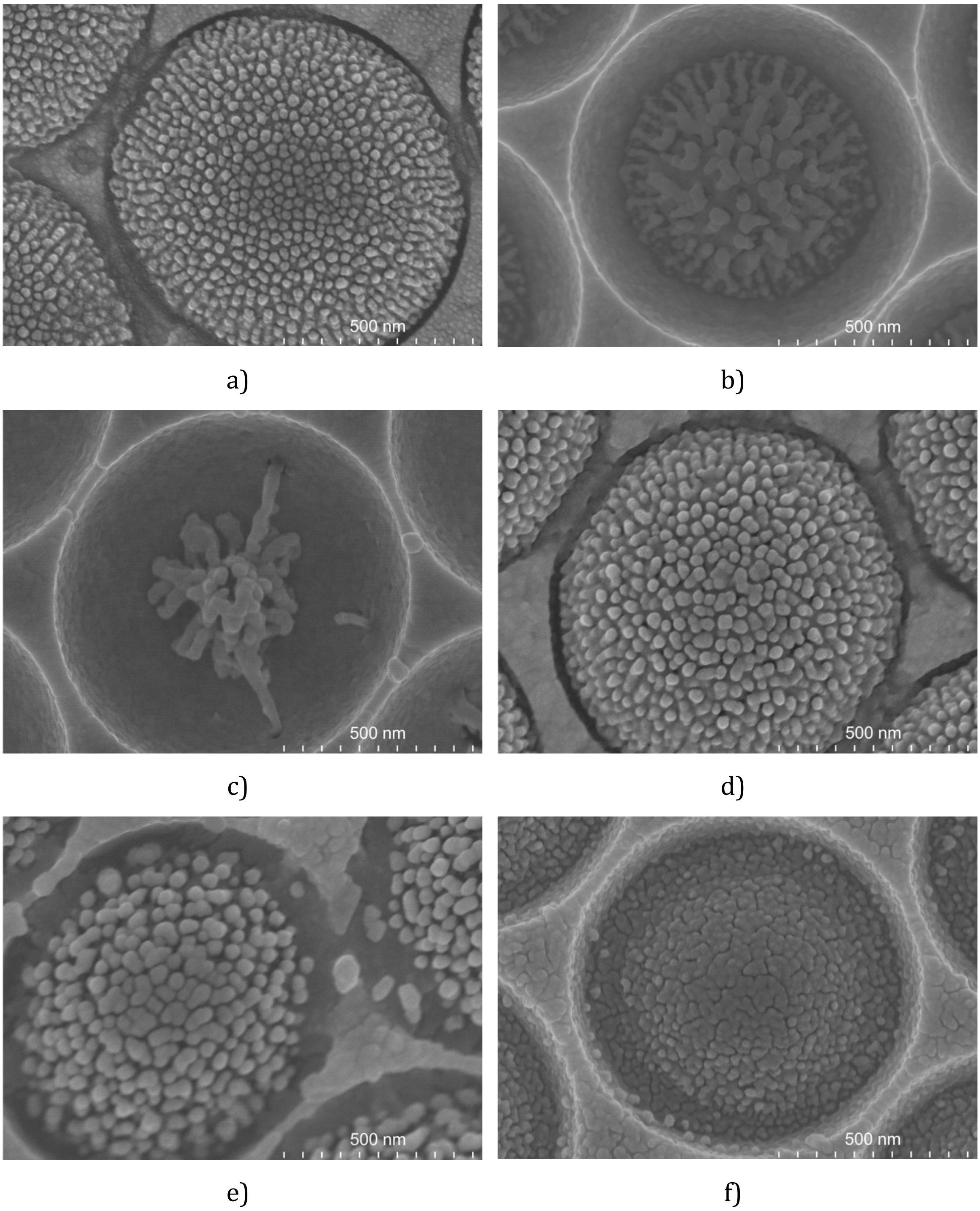

Figure S 6 : : Representative FESEM images of 1 µm diameter PS sphere in cavity etched under different conditions. a) RF Power = 100 W, P = 50 mTorr, O_2 = 25 sccm; t = 1 min; b) RF Power = 100 W, P = 50 mTorr, O_2 = 25 sccm; t = 5 min; c) RF Power = 100 W, P = 50 mTorr, O_2 = 25 sccm; t = 10 min; d) RF Power = 100 W, P = 50 mTorr, O_2 = 50 sccm; t = 1 min; e) RF Power = 200 W, P = 50 mTorr, O_2 = 25 sccm; t = 1 min; f) RF Power = 100 W, P = 50 mTorr, Ar = 50 sccm; t = 10 min.

2.2 Etching 3 µm diameter PS sphere with RF Power of 200 W

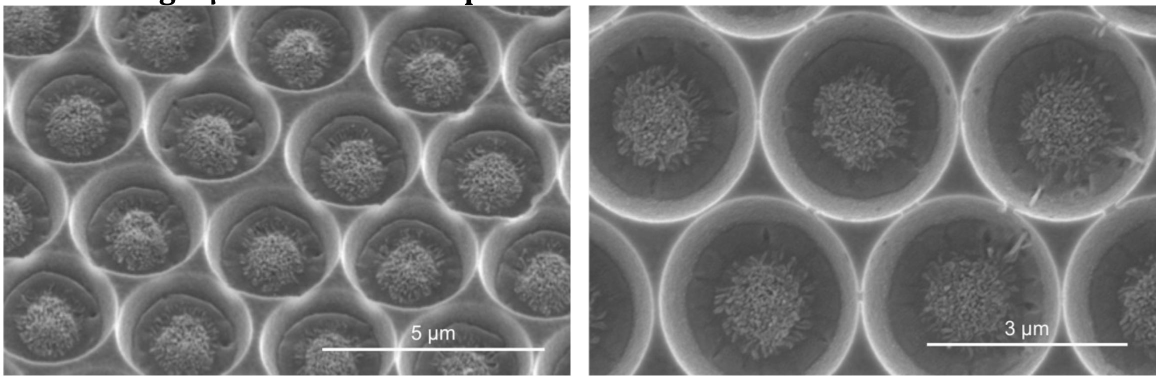

Figure S 7 : SEM images of a "water lily" shaped particle resulting from a 3 µm diameter PS spheres in cavity etched for 15 min with a RF power of 200 W.

2.3 icNP diameter vs. etching time during etching of 510 nm diameter PS sphere with RF Power of 50 W

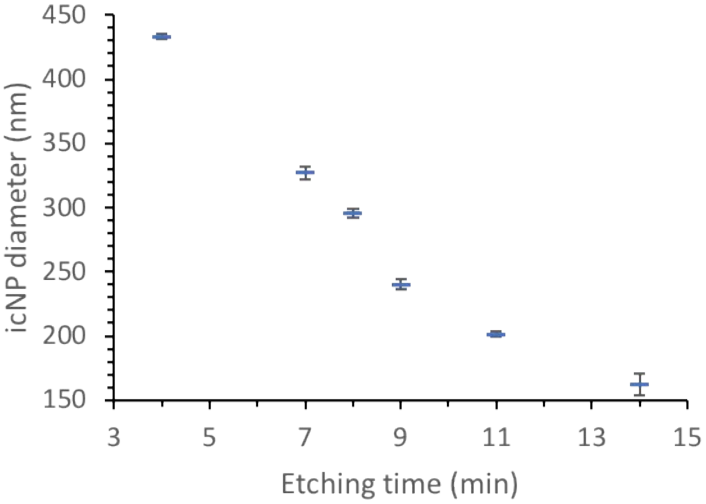

Figure S 8 : icNPs diameter vs. etching time during etching of 510 nm diameter PS sphere (RF power = 50 W; P = 50 mTorr; O_2 = 25 sccm).

3 Importance of a highly uniform monolayer PS sphere for a uniform distribution of icNPs

3.1 Defects created when etching multilayer of polymer

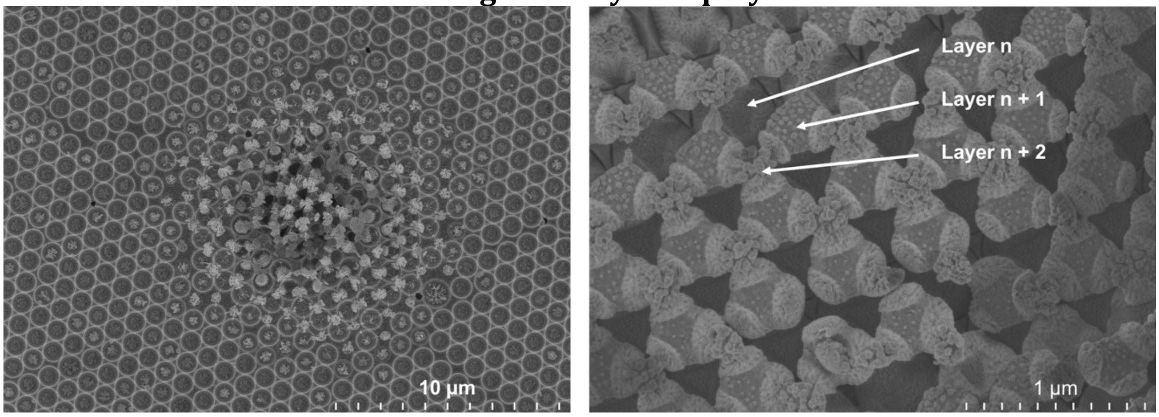

Figure S 9 : FESEM images of defects created by etching multilayers assembly of PS spheres at the superficial interstitial space of 1 µm (left) and 510 nm (right) diameter cavity arrays.

3.2 Tilted view of icNPs arrays after etching a highly uniform monolayer of PS spheres

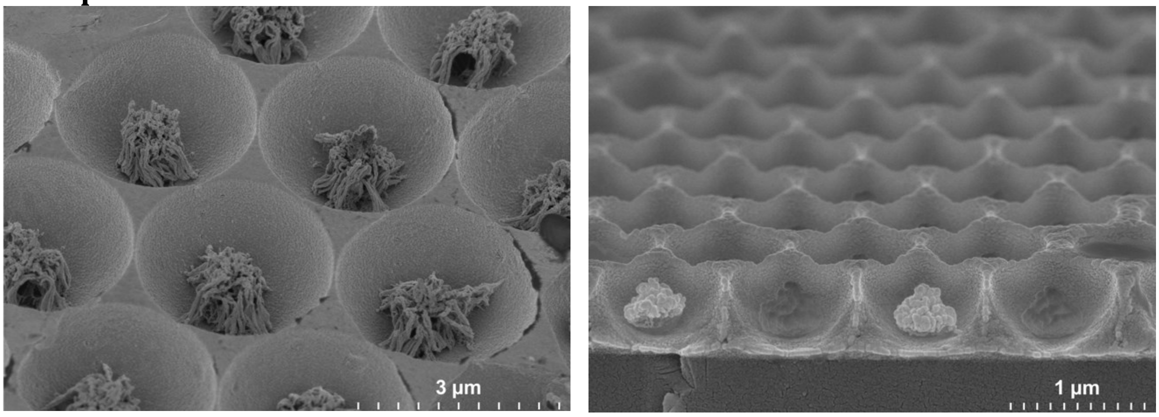

Figure S 10 : Tilted view FESEM images of a 3 µm diameter PS spheres in cavity etched for 50 min with a RF power of 100 W (left) and a 1 µm diameter PS spheres in cavity etched for 8 min with a RF power of 100 W

4 SERS enhancement factor detailed calculation

Analysing the relative Raman intensity of the ν_3 inter-ring stretching (B_2 ring) mode at 1293 cm^{-1}, SERS enhancement factors (EF_{SERS}) were estimated for the 10 mM ethanolic solution of 4,4'-BPY adsorbed at the Au substrates with and without icNPs, relative to 4,4'-BPY in its solid form according to the following equation:

$$EF_{SERS} = \frac{I_{SERS}}{I_{RS}} \times \frac{N_{RS}}{N_{SERS}}$$

where I_{RS} and N_{RS} are respectively the Raman signal intensity and the corresponding number of molecules for 4,4'-BPY in its solid form, and I_{SERS} and N_{SERS} the SERS signal intensity and the corresponding number of molecules at the SERS substrate's surface.

We estimated N_{RS} as follows:

$$N_{RS} = \frac{V_{exc} \times \rho_{4,4' - BPY} \times N_A}{MW_{4,4' - BPY}}$$

Where :

- the optical excitation volume $V_{exc} = \pi \times r^2 \times h$

  with the laser spot radius $r = 0.61 \times \frac{\lambda_{exc}}{N.A.}$

  and the depth of focus $h = 2 \times \frac{\lambda_{exc}}{N.A.^2}$

  (Excitation wavelength $\lambda_{exc} = 785\ nm$, Numerical aperture $N.A. = 785\ nm$)
- the density of 4,4'-BPY $\rho_{4,4' - BPY} = 1.232$ at room temperature. Value calculated based on unit cell and atoms in molecule from crystallography data base.(1)
- the Avogadro number $N_A = 6.022140857 \times 10^{23}\ mol^{-1}$
- the molecular weight of 4,4'-BPY $MW_{4,4' - BPY} = 156.19\ g.mol^{-1}$

To estimate N_{SERS}, we first approximate the surface area available for functionalisation of 1 unit cell for cavities without and with icNPs. Considering a hexagonal close packed arrangement of hemispherical gold cavities on the SERS substrates, the surface of a unit cell was calculated using the nominal diameter of the PS spheres used to fabricate the arrays. In this type of arrangement, a unit cell consists of 1 cavity and 2 interstitial triangles. For cavities with icNPs, correlating morphology characterised by FESEM to SERS performance as a function the NPs optimisation through variation of the etching time, we estimate that best SERS enhancement is obtained when the ratio η (icNPs diameter / cavity diameter) is around 0.4, corresponding to a NP's height of circa two-third of the cavity's height. Based on those dimensions, icNPs were assimilated to 3D-shapes formed by a cone mounted onto a spherical cap at the base fitting perfectly to

the bottom of the cavity (i.e. shape of an inverted one scoop ice-cream cone). The corresponding surface area of a unit cell could then be estimated.

Number of molecules in 1 unit cell ($N_{unit\ cell}$) was then estimated assuming:

- 4,4'-BPY molecules were adsorbed as a monolayer, as samples were rinsed with a large volume of ethanol after overnight functionalisation to ensure physi-sorbed molecules do not contribute to the SERS signal;
- 100% adsorption efficiency leading to full coverage of the available surface area;
- Surface area of a single 4,4'-BPY molecule was estimated to be 0.4 nm^2.(2)

Finally, N_{SERS} was estimated as follows:

$$N_{SERS} = N_{Unit\ cell} \times \frac{A_{Laser\ spot}}{A_{Unit\ cell}}$$

Where:

- the laser spot size $A_{Laser\ spot} = \pi \times r^2$
- the planimetric area of 1 unit cell $A_{Unit\ cell} = d_{cav}^{\ 2} \times \frac{\sqrt{3}}{2}$, with d_{cav} the diameter of 1 cavity.

5 Reusable microfluidic device for SERS and MEF application

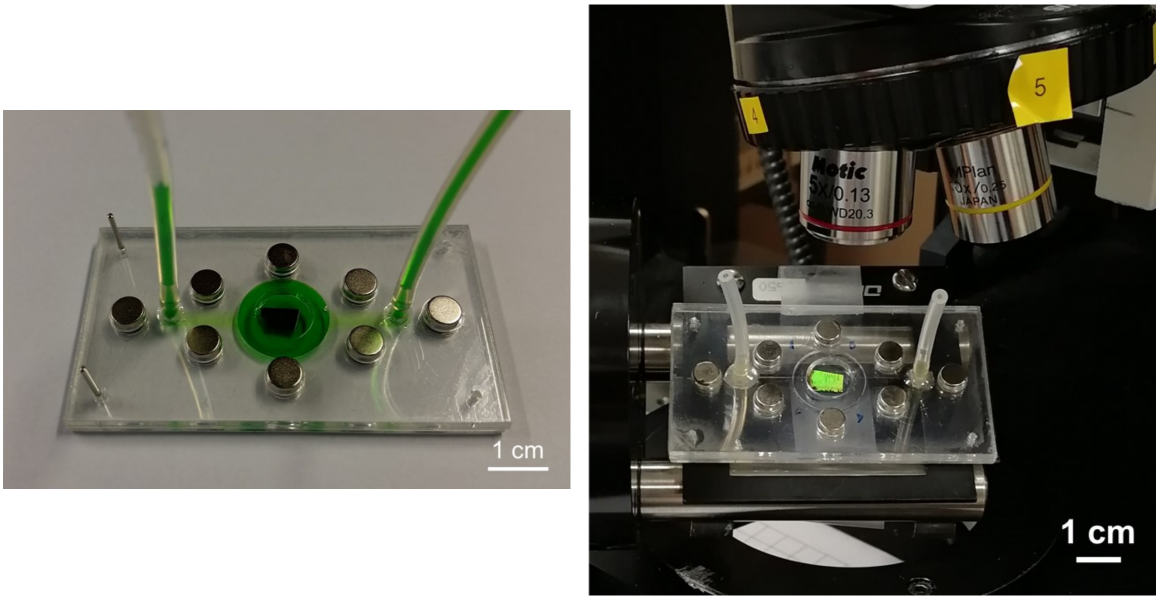

Figure S 11 : Reusable PMMA based microfluidic device designed for SERS and MEF measurement in liquid. (Left) The green liquid is a food dye used to test the sealing

efficiency of the system. (Right) 1 µm diameter cavity arrays with icNPs in water for SERS measurement.

6 References

1. Candana MM, Eroĝlu S, Özbeya S, Kendi E, Kantarci Z. Structure and Conformation of 4,4′-Bipyridine. Spectroscopy Letters. 1999 Jan;32(1):35–45.

2. Suzuki M, Niidome Y, Yamada S. Adsorption characteristics of 4,4′-bipyridine molecules on gold nanosphere films studied by surface-enhanced Raman scattering. Thin Solid Films. 2006 Feb 21;496(2):740–7.
